# Mathematics performance predicts cognitive and affective math anxiety through mutual mediation pathways from adolescence onward with potential working memory moderations

**DOI:** 10.1038/s41598-026-45516-y

**Published:** 2026-03-29

**Authors:** Chin-Yuan Chang, Min Hsiao, Wen-Chi Chiang

**Affiliations:** Department of Psychology, National Chung Cheng University, Chiayi, 621301 Taiwan (R.O.C.)

**Keywords:** Mathematics anxiety, Math performance, Phonological working memory, Visuospatial working memory, Moderated mediation, Neuroscience, Psychology, Psychology

## Abstract

This research investigated the pathways from math performance to the cognitive (worried thoughts) and affective (emotional tension) dimensions of math anxiety (MA) in high-school and university students. Using the PLS-SEM method, we identified a consistent dual-pathway mediation structure across both groups: Math performance is associated with affective MA via cognitive MA, and with cognitive MA via affective MA. We also explored whether working memory (WM) subsystems, based on Baddeley’s model, moderate these links and found differences between these two successive academic cohorts. In high-school students, phonological loop (verbal) WM moderated the link from math to affective MA, with this effect being evident only for those with medium-to-low capacity. In contrast, for university students, a dual-pathway moderated-mediation structure was found: the pathway to cognitive MA was contingent upon medium-to-high capacity in visuospatial sketchpad (representing image and location) WM, whereas the pathway to affective MA was contingent upon medium-to-high capacity in both phonological and visuospatial WM. However, simpler mediation models without WM interactions consistently showed superior model fit. These findings support a stable interplay between the MA components across the sampled academic stages, emphasizing the explanatory and predictive primacy of these core mediation mechanisms during the transition from adolescence to young adulthood.

**Supplementary Information:**

The online version contains supplementary material available at 10.1038/s41598-026-45516-y.

## Introduction

Mathematics proficiency is fundamental to individual and societal success, correlating with long-term socioeconomic outcomes and advancements in science and engineering^1,2^. Its inclusion as a core subject in compulsory education underscores its importance. However, mathematics performance has long been associated with mathematics anxiety (MA), defined as feelings of tension and apprehension arising from experiences with mathematics. A substantial body of research, including meta-analyses, consistently confirms a negative relationship between MA and mathematics achievement^3,4^.

While the influence of MA on performance is well-established, recent research has begun to explore the possibility of a reverse link, wherein poor mathematics performance may also precipitate or intensify MA. Some studies have provided supporting evidence^5,6^, but more thorough investigation of this specific causal direction—from poor performance to increased anxiety—is still needed to gain a full understanding of the reciprocal relationship. This knowledge is critical for designing comprehensive interventions that target both the cognitive and emotional barriers to mathematics learning.

### Definition and measurement of mathematics anxiety

According to a widely-cited early study on MA, MA refers to an individual’s feelings of tension or nervousness in daily-life or academic situations involving mathematics, which interfere with the ability to use numbers and solve mathematical problems, thereby negatively affecting mathematics achievement^7^. Research has consistently confirmed that MA is distinct from general and test anxiety, explaining unique variance in mathematics performance that cannot be attributed to other forms of anxiety^4,8–10^. However, while the traditional definition of MA remains relevant, recent literature has refined its conceptualization, shifting from a unidimensional view to a multidimensional construct involving specific cognitive and affective components^9,11,12^.

Recent empirical evidence continues to support the framework originally proposed by Wigfield and Meece, which distinguishes between an affective component (nervousness, tension, and physiological reactions) and a cognitive component (worry and intrusive thoughts about failure)^13^. This two-factor structure (cognition and affect) was recently validated across genders and populations^14^. Crucially, separating these dimensions is vital for understanding the MA–performance link, as recent findings suggest that the cognitive dimension (worry) often serves as a stronger predictor of performance deficits than the affective dimension alone^15^.

In terms of assessment, although the Mathematics Anxiety Rating Scale was historically significant, it treated MA as a unidimensional construct^7^, as did the Mathematics Attitude Scales^16^. To accurately capture the distinct components and their specific relationships with mathematics performance, instruments that explicitly distinguish between dimensions are required. Consequently, the Mathematics Anxiety Questionnaire (MAQ) was employed in the current research as a representative instrument developed based on this multidimensional framework to separately assess the cognitive and affective dimensions of MA^13^.

### The relationship between mathematics anxiety and mathematics achievement

The relationship between MA and mathematics achievement has been a central topic in educational research. Meta-analyses consistently confirm a significant, negative relationship between them across diverse student populations^9,11,15^. While early theories debated the causal directions, a consensus on reciprocal models of the link is emerging, supported by longitudinal data demonstrating that MA and achievement engage in a vicious, mutually reinforcing cycle^5,6,17^.

However, the understanding of the psychological mechanisms underlying the two directional relationships differs in depth. The mechanisms driving the anxiety-to-achievement pathway are well-documented by cognitive interference theory, where worrying consumes finite working memory (WM) resources and in turn impairs performance^18^. In contrast, the reversed pathway is typically explained by frameworks such as the deficit theory (i.e., awareness of low math performance elevates MA^17^) or the reciprocal theory^5^, but unlike the interference model, the specific psychological mechanisms driving the impact of past performance on future anxiety remain less delineated.

To account for the predictive link from achievement to MA, Pekrun’s^19,20^ Control-Value Theory (CVT) offers a comprehensive framework in which specific mechanisms drive the achievement-to-MA relationship, that is, the academic outcomes shape the students’ emotions through their appraisals of control and value. This framework supports two plausible dimensional pathways regarding the temporal sequence of anxiety components. On the one hand, CVT suggests that failure primarily diminishes a student’s sense of control (a cognitive appraisal), which then triggers negative emotional arousal^20,21^. Longitudinal findings substantiate this in the mathematics domain, showing that achievement predicts control appraisals, which in turn predict achievement emotions^22^. Thus, academic setbacks may first instigate maladaptive control appraisals, which subsequently elicit the cognitive and physiological components of anxiety. On the other hand, the theory also recognizes reciprocal causation where emotional experiences shape subsequent appraisals. For instance, an immediate negative emotional reaction (affective MA) can activate mood-congruent memories and reduce perceived control, thereby fostering persistent worry (cognitive MA)^20,21^.

In a recent cross-sectional study, Henschel and Roick^23^ found evidence for both pathways, and hypothesized the existence of a mutual relationship. This hypothesis, however, has not been formally tested, and the causal dynamics of interactions among math achievement and MA dimensions remain unspecified and require further investigation.

### The role of working memory in the relationship between mathematics anxiety and mathematics achievement

WM is a critical cognitive mechanism implicated in the MA–achievement relationship. According to Baddeley’s multi-component model, WM is not a unitary system but consists of a “central executive”—a domain-general control system—supported by two domain-specific storage buffers: the “phonological loop,” which processes verbal and auditory information, and the “visuospatial sketchpad,” which handles visual imagery and spatial orientation^24^. This framework is critically important in the context of mathematics learning, as different mathematical tasks rely on the subsystems differentially. For instance, the phonological loop is essential for counting, maintaining intermediate steps in mental arithmetic, and retrieving arithmetic facts, while the visuospatial sketchpad is crucial for representing magnitudes, understanding geometry, and manipulating mental number lines^24,25^.

In the well-documented pathway from MA to achievement, WM is understood to function as a key mediator. Consistent with Attentional Control Theory (ACT)^26^, the intrusive thoughts characteristic of MA—particularly the cognitive dimension (worry)—are thought to primarily preempt the finite resources of the central executive. According to ACT, this creates a dual-task environment where worry competes with task-related processing. Because the central executive is responsible for coordinating the auxiliary phonological and visuospatial systems and managing attentional functions such as inhibition and shifting, its occupation by math-related worry would reduce processing efficiency on mathematical tasks. Consequently, fewer resources remain available for complex mathematical execution, leading to impaired performance^27^.

Beyond its role as a mediator, the function of WM as a moderator is a subject of considerable theoretical debate, centering on two competing accounts. One account posits that high WM capacity serves as a protective buffer^28^, and accordingly, individuals with greater WM resources are less susceptible to the effects of anxiety because they possess a larger “reserve capacity” to deploy for task execution. Conversely, an alternative account proposes that high WM capacity can be a vulnerability^29,^ as detrimental effects of MA are likely more pronounced in such individuals because of their strong tendency, relative to that of low WM-capacity individuals, to adopt strategies engaging either visuospatial^30^ or phonological^31^ WM subsystems in solving math problems.

In stark contrast, the role of WM in the reciprocal direction—how prior achievement influences subsequent MA—remains underexplored. Theoretically, WM could moderate this performance-to-MA pathway as well. High WM capacities, for instance, may provide the cognitive architecture for academic resilience, enabling top-down regulatory functions^32^ to reframe setbacks. Conversely, lower WM capacities could exacerbate an individual’s vulnerability to developing MA following repeated difficulties^33^. Given their distinct roles in mathematical processing, the visuospatial and phonological components of WM may also differentially moderate the achievement-to-MA link. Exploring these specific dynamics is essential for explaining why some high-ability students develop debilitating MA while others remain resilient.

### The present research

The present research had two primary goals. First, building upon Henschel and Roick’s^23^ foundational work with elementary-school children in a western culture, our studies aimed to expand the understanding of the achievement-to-MA link by applying the two-dimensional MA conceptualization using the MAQ^13^ to high-school (Study 1) and university students (Study 2) within a non-western cultural context. To achieve this, we incorporated measures of math achievement, of cognitive and affective dimensions of MA, and of capacities of WM subsystems, phonological loop and visuospatial sketchpad (hereafter “phonological” WM and “visuospatial” WM for convenient references, respectively).

To evaluate the relationships among these constructs, we tested two competing mediation pathways: Model CA (math performance [Perf] → cognitive MA [cMA] → affective MA [aMA]) and Model AC (Perf → aMA → cMA). Relevant demographic and academic factors were included as methodological controls in all analyses. Previous findings with elementary students suggest that the relationship between the two MA components is bidirectional^23^. Moreover, while the CVT highlights appraisals as antecedents of emotions, it explicitly posits a feedback loop where emotions reciprocally influence appraisals^19^. Therefore, to examine whether these patterns extend to older students, we hypothesized a bidirectional relationship wherein math performance influences MA through both cognitive and affective pathways (Hypothesis 1). We further conducted model comparisons as an empirical inquiry to determine if one pathway (Model CA or Model AC) demonstrates stronger explanatory power than the other in each of the two student populations studied.

The second goal of this research was to explore the potential moderating roles of distinct WM components (phonological and visuospatial). This investigation was primarily exploratory, as the role that WM plays in the specific pathways from performance to MA has not been previously established. However, extending the “protective buffer” account^28^—which suggests that high WM capacity can mitigate the interference of anxiety on performance—we tentatively hypothesized that higher WM capacities would similarly act as a buffer in the reverse direction, dampening the anxiety-inducing effects of poor performance (Hypothesis 2). Nevertheless, given the lack of prior empirical evidence and the existence of the competing “vulnerability” account^29^, we remained open to alternative outcomes.

## Method

### Study 1

#### Participants

To determine the minimally required sample size for Partial Least Squares Structural Equation Modeling (PLS-SEM), we employed the minimum R-squared method with a weak *R*^2^ value of 0.25. While *R*^2^ = 0.25 may be considered moderate in general linear regression, it is categorized as the benchmark for “weak” explanatory power within PLS-SEM-specific evaluation guidelines^34^. Using the G*Power software version 3.1.9.7^35^, we calculated that for a weak *R*^2^ (which corresponds to a Cohen’s^36^ effect size *f*^2^ = *R*^2^/(1—*R*^2^) = 0.33) with 9 exogenous variables, a sample size of 57 is needed to achieve an 80% power at the 5% significance level. A total of 65 junior and senior high-school students (32 males and 33 females; mean age = 15.68 years, *SD* = 0.5) in Taiwan were recruited. All participants had normal or corrected-to-normal vision and hearing, and no color blindness. They were native speakers proficient in reading and writing Traditional Chinese. To control for potential learning differences, participants were recruited from general education classes and reported no history of diagnosed learning disabilities or special educational needs.

#### Materials

**Math anxiety** MA was assessed using the Chinese version of the MAQ^13^. We adopted 9 items from the original 11-item scale based on the study by Ho et al. ^37^, which applied the MAQ to sixth-grade elementary-school children from various regions, including Taiwan, and found an improved model fit after removing two certain items. Among the 9 items adopted for the present study, five measured the affective component, and four measured the cognitive component of MA. A 7-point Likert scale was used.

As Ho et al. ^37^ administered the Chinese version of MAQ to sixth-graders exclusively, we assessed its applicability to adolescents by testing a group of junior high-school students (*N* = 142) and conducting a confirmatory factor analysis (CFA) using the lavaan package^38^. The results indicated an acceptable model fit (Comparative Fit Index [CFI] = 0.97, Non-Normed Fit Index [NNFI] = 0.96, Root Mean Square Error of Approximation [RMSEA] = 0.08, and Standardized Root Mean Square Residual [SRMR] = 0.06), meeting established criteria (i.e., CFI/NNFI > 0.95, SRMR < 0.08^39^; RMSEA < 0.08^40^), and supported a two-factor structure corresponding to the affective and cognitive components of MA. Although it has been recommended by some researchers to use the value 0.708 as a criterion^34^, factor loadings are often not that high in social science research, and loadings exceeding 0.55 are generally considered practically significant^41^. The analysis revealed that the standardized factor loadings ranged from 0.57 to 0.94 for the affective factor and from 0.63 to 0.93 for the cognitive factor, all exceeding 0.55. The Average Variance Extracted (AVE) was 0.64 for the affective, and 0.59 for the cognitive dimension, both exceeding the 0.50 threshold. Composite reliability was 0.89 for the affective, and 0.85 for the cognitive dimension, both above the 0.60 criterion, indicating good convergent validity^42^. In addition, the heterotrait-monotrait ratio of correlations (HTMT) was 0.81, below the 0.85 threshold^43^, demonstrating adequate discriminant validity. Overall, these results suggest that the 9-item Chinese version of MAQ is suitable for use with secondary-school students.

**Working memory** WM was assessed using two tasks targeting the visuospatial and phonological components, respectively. Following Baddeley’s theoretical framework^24^, we measured the capacities of these WM subsystems using different types of stimuli with an identical backward-recall procedure, to eliminate variance associated with procedural differences. The tasks were programmed and administered online using Labvanced^44^. Participants completed the tasks by giving their responses with the keyboard and mouse at their end after the experimenter explained the procedure via Google Meet.

For the visuospatial WM task, each trial began with a fixation cross presented at the center of the screen, followed by a sequence of 4 × 4 grids in each of which one cell contained a black dot. Each grid was displayed for 750 ms, followed by a 250-ms blank screen. The sequence continued until all grid displays for the trial had been presented. A blank 4 × 4 grid then appeared, and the participants were instructed to mouse-click on the positions of the black dots seen on that trial in reverse order. The clicked positions in the grid were visually indicated on screen as input for the trial. Three buttons were presented on screen along with the blank grid, allowing the participant either to “skip” the specific position in a sequence when that position could not be recalled, or to “clear” the lastly clicked position and modify the input, before clicking “submit” to finalize the input for the trial and initiate the next trial. In any given trial, the black dots were never presented more than once in any cell of the 4 × 4 grid.

For the phonological WM task, the auditory stimuli consisted of words for digits from 2 to 9 in Mandarin (all were one-syllable words; digit 1 was excluded as its pronunciation in Mandarin was highly similar to that of digit 7). All digit recordings were synthesized using Google and Azure text-to-speech systems. Each trial began with a fixation cross, followed by a 750 ms audio recording of a digit and a subsequent 250-ms silent interval. This process continued until all digits for that trial had been presented. A white response area then appeared at the center of the screen, and the participants were instructed to mouse-click the digit buttons on the screen in reverse order of the digit string heard on that trial. The participants’ digit string responses appeared in the response area in a left to right direction, and the same three response buttons were available for use as in the visuospatial task before one trial was completed and the next initiated. In any given trial, no digit appeared more than twice, and the same digit was never presented consecutively.

For both WM tasks, each memory span level (i.e., the number of items to be remembered) included two trials. The starting span level was set at two for the visuospatial task and at three for the phonological task, according to our pilot data on the average minimum span at which both trials were correctly answered by the participants. The procedure was as follows: At a given span level, if the first trial was answered correctly, the span level on the next trial increased by one, until reaching the maximum span level of 16. If the first trial was answered incorrectly, the second trial at the same span level was administered. At a span level where the participants failed both trials, the task proceeded to present the second trial at the span level decreased by one, until the participant either successfully recalled both trials at the same level, or completed both trials at the minimum span level of one.

To ensure a sufficient understanding of task procedures, the participants received four practice trials prior to each of the WM tasks, which presented test items that were at a low span level and distinctly different from those in the task proper. At the end of each practice trial, a display appeared on screen briefly to indicate whether the response was correct before automatically advancing to the next trial or the practice ended. No such feedback was provided during the task proper. For the phonological WM task, the recordings for all the eight digits (2 to 9) were included in the practice trials to ensure that participants could clearly hear and correctly identify each of them.

The WM task procedure we adopted was designed to efficiently estimate both the highest span level at which the participant correctly answered in both trials and the lowest span level at which the participant failed both. The scoring method was identical for both WM tasks and as follows: For span levels below the highest level with two correct responses, one point was assigned per level; for span levels above the lowest level with two incorrect responses, zero points were assigned; for the span levels in between, 0.5 points were assigned for each correct response. The sum of these values from each WM task represented the WM span scores for each participant.

**Math achievement** The standardized scores from the Comprehensive Assessment Program for Junior High School Students (CAP)—the nationwide senior high-school entrance examination in Taiwan—were used as indexes for math achievement. These standardized scores were selected to ensure comparability across participants and to maximize the detection of moderating effects by distinct types of WM, as such scores reflect an overall proficiency of various math skills and the exams were of great importance to all participants.

#### Procedure

Study 1 was reviewed and approved by the Human Research Ethics Center of National Chung Cheng University. All methods were performed in accordance with the relevant guidelines and regulations, including the Declaration of Helsinki. All participants provided written informed consent before participating in the study.

We employed a sequential measurement approach where the administration of MA measures was temporally distinct from the earlier assessment of math achievement. This design ensured that MA reports were not influenced by any immediate math task performance in the definite temporal albeit not necessarily causal sequence. The participants first completed the visuospatial and phonological WM tasks, and then the MAQ. They also provided their CAP score reports—the standardized senior high-school entrance examination in Taiwan—from which the mathematics and Chinese language scaled scores (range: 1 to 7) were extracted to represent each participant’s math achievement and Chinese language proficiency, respectively.

#### Data analysis

We used PLS-SEM to test the hypothesized pathways. PLS-SEM is a variance-based approach that is particularly well-suited for estimating complex path relationships and mediation effects within a single integrated model^34^. Compared to the hierarchical regression approach used in previous work^23^, PLS-SEM provides a more robust framework for testing mediation and is effective even in studies with relatively small sample sizes^34^. We used the SEMinR package^45^ in R 4.4.2^46^ to perform PLS-SEM. Gender and language proficiency were included as methodological controls, as previous research has identified them as potential correlates of MA and achievement^11,47^. The year of exam participation was also included as a methodological control to account for potential variance arising from different test items across cohorts, even when standardized scores were used. We examined the mediation model by a mediation analysis and the moderated mediation model by a conditional mediation analysis^48^ along with a Johnson-Neyman analysis to illustrate the conditional effect at different standardized levels of the moderator. All analyses involving statistical inference were based on bootstrapping with 5,000 resamples. Effects were deemed statistically significant if their 95% bootstrap CIs did not contain zero.

The measurement model was evaluated by assessing internal consistency reliability (Cronbach’s alpha, Composite Reliability [rho_C_], and rho_A_ > 0.70), convergent validity (AVE > 0.50), and factor loadings (> 0.50). Discriminant validity was confirmed via the HTMT, an index that estimates the true correlation between latent constructs. Following established guidelines^43^, we adopted a conservative threshold of 0.85 for distinct constructs and a threshold of 0.90 for conceptually similar constructs (i.e., the affective and cognitive dimensions of MA) to indicate adequate discriminant validity. Collinearity was monitored using variance inflation factor (VIF) values, with values below 5 indicating no issues. Next, the structural model was assessed to determine the significance of direct paths, mediation effects, and the moderated mediation effects, as well as the model’s explanatory power (*R*^2^) and effect sizes (*f*^2^). *R*^2^ values represent the variance explained in the endogenous latent factors; following the PLS-SEM-specific guidelines^34^, values of 0.75, 0.50, and 0.25 are considered substantial, moderate, and weak, respectively. Regarding *f*^2^, values of 0.02, 0.15, and 0.35 denote small, medium, and large effects^36^. Crucially, for moderation effects, specific thresholds of 0.005, 0.010, and 0.025 are used to represent small, medium, and large contributions to the endogenous construct’s explanation^34^.

Model comparisons were conducted using the Bayesian Information Criterion (BIC) to select the most parsimonious model. BIC was selected because it effectively balances model fit and complexity, providing a robust metric for model selection in PLS-SEM that is effective even with smaller samples where splitting the data into training and testing sets (a holdout sample) is not feasible^49,50^. Subsequently, the out-of-sample predictive power of the selected model was assessed using PLS_predict_ (10 folds, 10 repetitions). Predictive performance was evaluated using the Root Mean Square Error (RMSE), appropriate under symmetric error distributions, by comparing PLS-SEM results with those of a Linear Model (LM) benchmark^34^. High predictive power is indicated when all PLS-SEM manifest indicators (items) yield lower RMSE values than the LM; medium power when the majority are lower; and low power when the minority are lower^51^.

### Study 2

#### Participants

A group of 64 university students (32 males and 32 females; mean age = 21.7 years, *SD* = 0.97) were recruited in Taiwan to participate in the study. Inclusion criteria were identical to Study 1: participants were required to have normal or corrected-to-normal vision and hearing, no color blindness, and proficiency in Traditional Chinese. Furthermore, all participants confirmed having no history of diagnosed learning disorders or neurological conditions.

#### Materials

**Math anxiety** The assessment used to measure MA was 9-item Chinese version of the MAQ^13^. We assessed its applicability by testing a group of university students (*N* = 153) and conducting a CFA. The CFA results indicated an acceptable model fit (CFI = 0.97, NNFI = 0.96, RMSEA = 0.08, and SRMR = 0.04), meeting established criteria (i.e., CFI/NNFI > 0.95, SRMR < 0.08^39^; RMSEA < 0.08^40^), and supported a two-factor structure corresponding to the affective and cognitive components of MA. The standardized factor loadings ranged from 0.71 to 0.86 for the affective factor and from 0.62 to 0.79 for the cognitive factor. All loadings exceeded 0.55, which is considered practically significant in social science research^41^. The AVE was 0.64 for the affective dimension and 0.54 for the cognitive dimension, both exceeding the 0.50 threshold. Composite reliability was 0.90 for the affective dimension and 0.82 for the cognitive dimension, both above the 0.60 criterion, indicating good convergent validity^42^. In addition, the HTMT was 0.88, below the 0.90 (i.e., conceptually similar constructs) threshold^43^, demonstrating adequate discriminant validity. Overall, these results suggest that the 9-item Chinese version of the MAQ is suitable for use with university students.

**Working memory** The WM tasks administered in Study 2 were identical to those used in Study 1.

**Math achievement** Identical to the rationale in Study 1, standardized scores from a nationwide academic entrance examination were used. Specifically, scores from the General Scholastic Ability Test (GSAT)—one of the major university entrance examinations in Taiwan—were extracted. These scores ensure comparability across participants and reflect an overall math proficiency in a high-stakes testing context.

#### Procedure

Study 2 was reviewed and approved by the Human Research Ethics Center of National Chung Cheng University. All methods were performed in accordance with the relevant guidelines and regulations, including the Declaration of Helsinki. All participants provided written informed consent before participating in the study. Consistent with the sequential measurement approach used in Study 1, the participants first completed the visuospatial and phonological WM tasks, and then the MAQ, to ensure MA was not influenced by immediate task performance. They also provided their score reports of the GSAT—one of the major university entrance examinations in Taiwan—from which the mathematics and Chinese language scaled scores (range: 0 to 15) were extracted to represent each participant’s math achievement and Chinese language proficiency, respectively.

#### Data analysis

The procedures used for data analyses were identical to those used in Study 1.

## Results

### Study 1

#### Mediation model and moderated mediation model

The average scores of affective and cognitive MA were 3.68 (*SD* = 1.38) and 4.58 (*SD* = 1.29), respectively. The mean span scores of phonological and visuospatial WM were 7.72 (*SD* = 2.17) and 4.93 (*SD* = 1.86), respectively. The mean scaled math score of the high-school entrance exam was 4.71 (*SD* = 1.62). Latent variable correlations are provided in Supplementary Table S4 and S8.

**Fig. 1:**
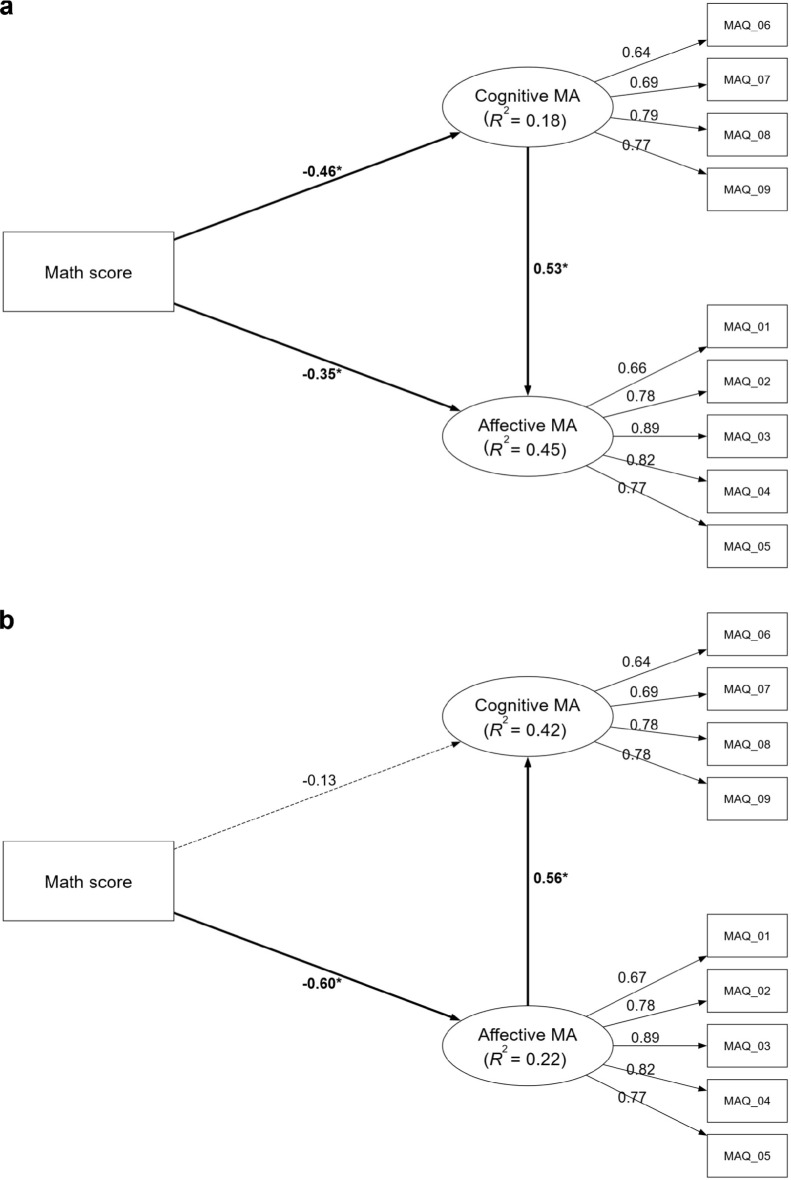
The mediation models of math anxiety in the adolescent sample. Panel (**a**) displays Model CA (cognitive MA [cMA] → affective MA [aMA]), testing the mediating role of cognitive math anxiety on the relationship between math performance and affective math anxiety. Panel (**b**) displays Model AC (aMA → cMA), testing the mediating role of affective math anxiety. Values on paths represent standardized beta coefficients ($\beta$). The R^2^ value under each endogenous construct indicates the proportion of its variance explained by the model. Significant paths are indicated by bold solid lines and marked with an asterisk (*), denoting that the bootstrapped 95% confidence interval (CI) excludes zero. Dashed lines indicate non-significant relationships. Detailed confidence intervals are provided in Supplementary Table S5. All models have controlled for gender, year of exam participation, and language score. MA: math anxiety; MAQ: items of the Math Anxiety Questionnaire.

The measurement model, identical across all analyses, demonstrated sound psychometric properties. All criteria specified in the Data Analysis section were met for internal consistency and convergent validity, with factor loadings surpassing 0.50 (see Fig. 1)^41^. All HTMT values were well below the more conservative threshold of 0.85, indicating strong discriminant validity^43^. All VIF values were below 5, indicating no multicollinearity issues (see Supplemental Results for details).

In the mediation analyses, Model CA (Perf → cMA → aMA) identified a partial mediation pathway (see Fig. 1a). A significant indirect effect ($\beta$ = -0.25, bootstrap 95% CI [-0.45, -0.10]) and total effect ($\beta$ = -0.60, bootstrap 95% CI [-0.86, -0.31]) indicate that lower math performance predicts higher cognitive MA, which in turn elevates affective MA. *R*^2^ values of 0.75, 0.50, and 0.25 are considered substantial, moderate, and weak, respectively ^34^. However, an *R*^2^ value as low as 0.10 may be deemed acceptable in social science research, provided that the majority of predictors are statistically significant^52^. The *f*^2^ indicates the impact of a predictor’s removal on an endogenous construct. Values of 0.02, 0.15, and 0.35 are regarded as small, medium, and large effects, respectively ^36^. The model explained limited variance in cognitive MA (*R*^2^ = 0.18, weak) but moderate variance in affective MA (*R*^2^ = 0.45). Effect sizes revealed the cognitive-to-affective link as the dominant mechanism (*f*^2^ = 0.41, large effect), whereas math performance had a medium effect on cognitive MA (*f*^2^ = 0.16) and a small direct effect on affective MA (*f*^2^ = 0.10).

Conversely, Model AC (Perf → aMA → cMA) supported a full mediation model (see Fig. 1b), with a significant indirect effect ($\beta$ = -0.33, bootstrap 95% CI [-0.56, -0.16]) and total effect ($\beta$ = -0.46, bootstrap 95% CI [-0.72, -0.20]). This suggests that lower performance predicts higher affective MA, which then increases cognitive MA. The model explained moderate variance in cognitive MA (*R*^2^ = 0.42) but only weak variance in affective MA (*R*^2^ = 0.22). The affective-to-cognitive path was dominant (*f*^2^ = 0.43, large effect), while math performance had a medium effect on affective MA (*f*^2^ = 0.28) and a negligible direct effect on cognitive MA (*f*^2^ = 0.01).

**Fig. 2:**
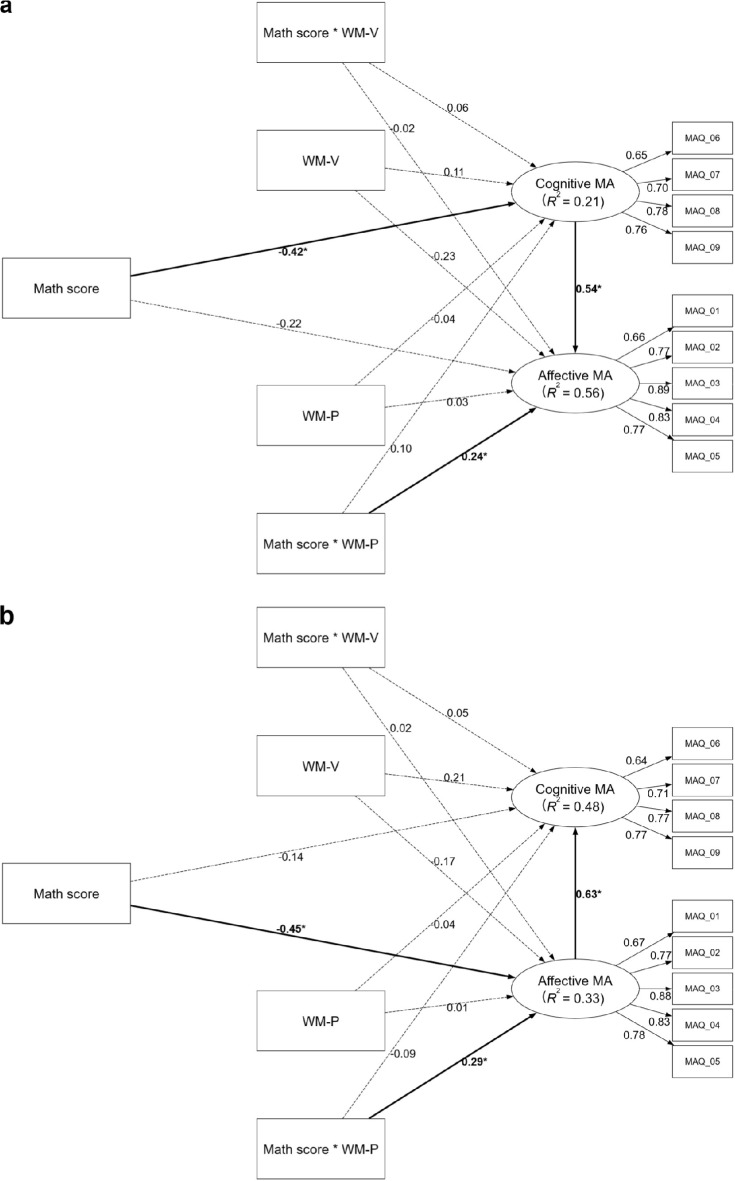
The moderated mediation models of math anxiety in the adolescent sample. Panel (**a**) displays Model CA (cognitive MA [cMA] → affective MA [aMA]), testing the mediating role of cognitive math anxiety on the relationship between math performance and affective math anxiety. Panel (**b**) displays Model AC (aMA → cMA), testing the mediating role of affective math anxiety. Both models also test the moderating roles of phonological (WM-P) and visuospatial (WM-V) working memory. Paths from interaction terms (e.g., Math score × WM-P) represent moderating effects, while paths from WM constructs represent their direct effects. Values on paths are standardized beta coefficients ($\beta$). The *R*^2^ value indicates the variance explained for each endogenous construct. Significant paths are indicated by bold solid lines and marked with an asterisk (*), denoting that the bootstrapped 95% confidence interval (CI) excludes zero. Dashed lines indicate non-significant relationships. Detailed confidence intervals are provided in Supplementary Table S9. All models have controlled for gender, year of exam participation, and language score. MA: math anxiety; WM-P: working memory (phonological); WM-V: working memory (visuospatial); MAQ: items of the Math Anxiety Questionnaire.

**Fig. 3:**
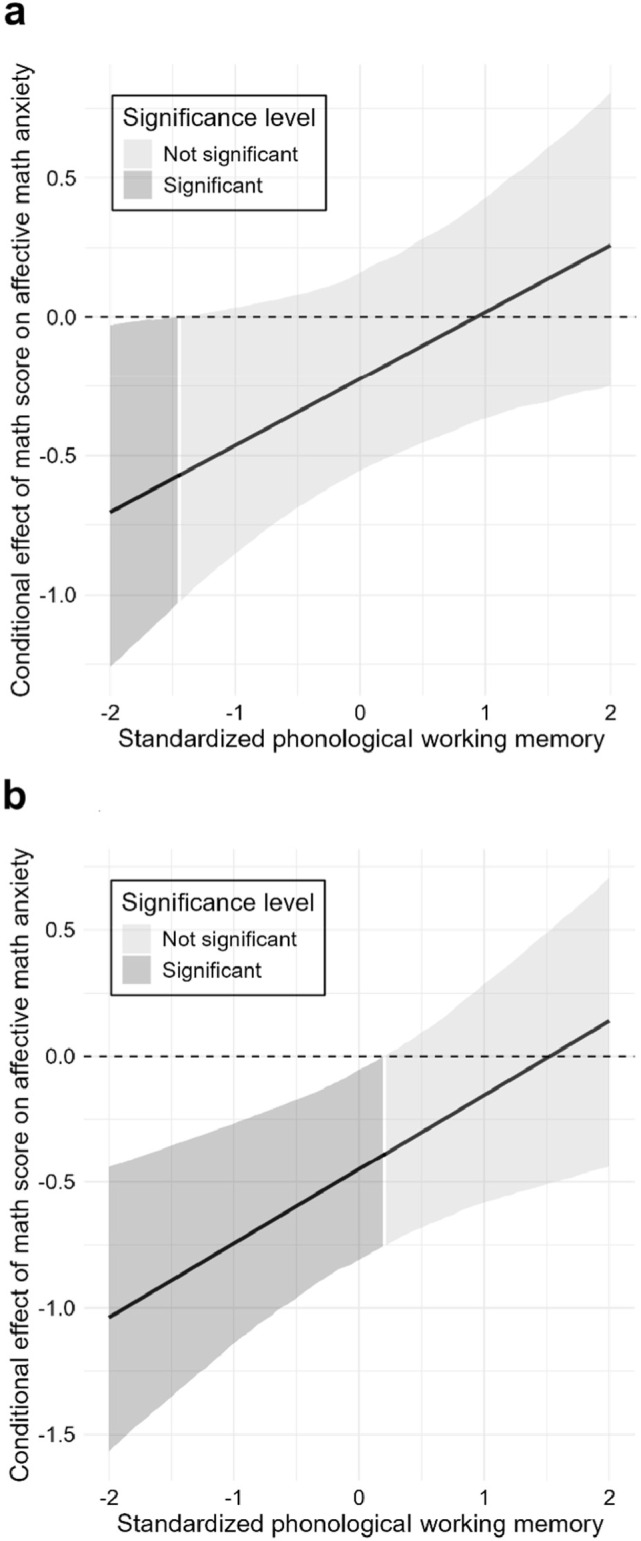
The moderating effects of phonological working memory in the adolescent sample. The Johnson-Neyman plots illustrate the conditional effect of math performance on affective math anxiety across standardized levels of phonological working memory. Panel (**a**) shows the moderating effect as estimated in Model CA (cognitive math anxiety [cMA] → affective math anxiety [aMA]), which tests the mediating role of cognitive math anxiety. Panel (**b**) shows the same effect as estimated in Model AC (aMA → cMA), which tests the mediating role of affective math anxiety. In both plots, the solid line represents the point estimate of the effect, with the surrounding shaded area indicating the 95% confidence interval. The dark gray region highlights where the effect is statistically significant.

In the moderated mediation analyses, Moderated Model CA did not support a moderated mediation effect (see Fig. 2a), though the significant indirect effect ($\beta$ = -0.23, bootstrap 95% CI [-0.48, -0.03]) and total effect ( $\beta$ = -0.45, bootstrap 95% CI [-0.81, -0.05]) persisted. Its explanatory power was moderate for affective MA (*R*^2^ = 0.56) and acceptable for cognitive MA (*R*^2^ = 0.21). For moderation effects, *f*^2^ values of 0.005, 0.010, and 0.025 represent small, medium, and large contributions to the explanation of the endogenous construct^34^. Phonological WM significantly moderated only the direct path from math performance to affective MA (*f*^2^ = 0.10, a very large effect), and the Johnson-Neyman analysis specified that the negative effect was significant only for individuals with low phonological WM (below -1.46 *SD*, see Fig. 3a). The moderating effect of phonological WM on the path to cognitive MA was non-significant (*f*^2^ = 0.01, medium effect). Visuospatial WM showed no moderating effects (*f*^2^s < 0.001).

Moderated Model AC, however, did support moderated mediation (see Fig. 2b), with a significant index of moderated mediation ($\beta$ = 0.18, bootstrap 95% CI [0.05, 0.37]), as phonological WM moderated the indirect pathway’s first stage. The significant indirect effect persisted ($\beta$ = -0.28, bootstrap 95% CI [-0.56, -0.03]), as did the total effect ($\beta$ = -0.42, bootstrap 95% CI [-0.78, -0.05]). The model’s explanatory power was weak-to-moderate for affective MA (*R*^2^ = 0.33) and moderate for cognitive MA (*R*^2^ = 0.48). The moderation was driven by the phonological WM on the performance-to-affective MA path (*f*^2^ = 0.10, a very large effect), which was significant for those with medium and low phonological WM (below 0.19 *SD*, see Fig. 3b). The moderating effect of phonological WM on the path to cognitive MA was non-significant (*f*^2^ = 0.01, medium effect). Visuospatial WM showed no effects (*f*^2^s < 0.003).

#### Model comparison and predictive power

To determine the optimal mediation sequence, BIC values were compared between competing models sharing the same outcome variable. When affective MA was the outcome, the mediation model specifying a path via cognitive MA (Model CA: Perf → cMA → aMA, BIC = -15.06) demonstrated a superior fit compared to the model where math performance led directly to affective MA (Model AC: Perf → aMA, BIC = 3.79). Conversely, when cognitive MA served as the outcome, the mediation model via affective MA (Model AC: Perf → aMA → cMA, BIC = -11.83) was statistically preferred over the direct path from math performance (Model CA: Perf → cMA, BIC = 6.99). Further model comparisons favored the more parsimonious mediation models as each was superior to their corresponding, more complex, moderated versions (Moderated Model CA, BIC = -12.64; Moderated Model AC, BIC = -1.58).

Following model selection, PLS_predict_ was conducted to assess out-of-sample predictive power, using RMSE as the metric. Model CA showed high predictive power for affective MA; its RMSE values (1.64, 1.68, 1.58, 1.35, 1.67) were consistently lower than the LM benchmarks (1.69, 1.76, 1.62, 1.37, 1.85). Model AC demonstrated medium predictive power for cognitive MA, with its RMSE values (1.45, 1.94, 1.54, 1.73) outperforming the LM benchmarks (1.66, 2.14, 1.48, 1.92) on three of four indicators.

In sum, these findings suggest two distinct mediation pathways from math performance to cognitive and affective MA, supporting their explanatory power and predictive utility without warranting added model complexity.

### Study 2

#### Mediation model and moderated mediation model

Study 2 tested undergraduate students as participants. The average scores of affective and cognitive MA were 3.95 (*SD* = 1.3) and 4.37 (*SD* = 1.34), respectively. The mean span scores of phonological and visuospatial WM were 7.9 (*SD* = 1.21) and 4.98 (*SD* = 1.08), respectively. The mean scaled math score of the university entrance exam was 10.31 (*SD* = 3.13). Latent variable correlations are provided in Supplementary Table S13 and S17.

**Fig. 4:**
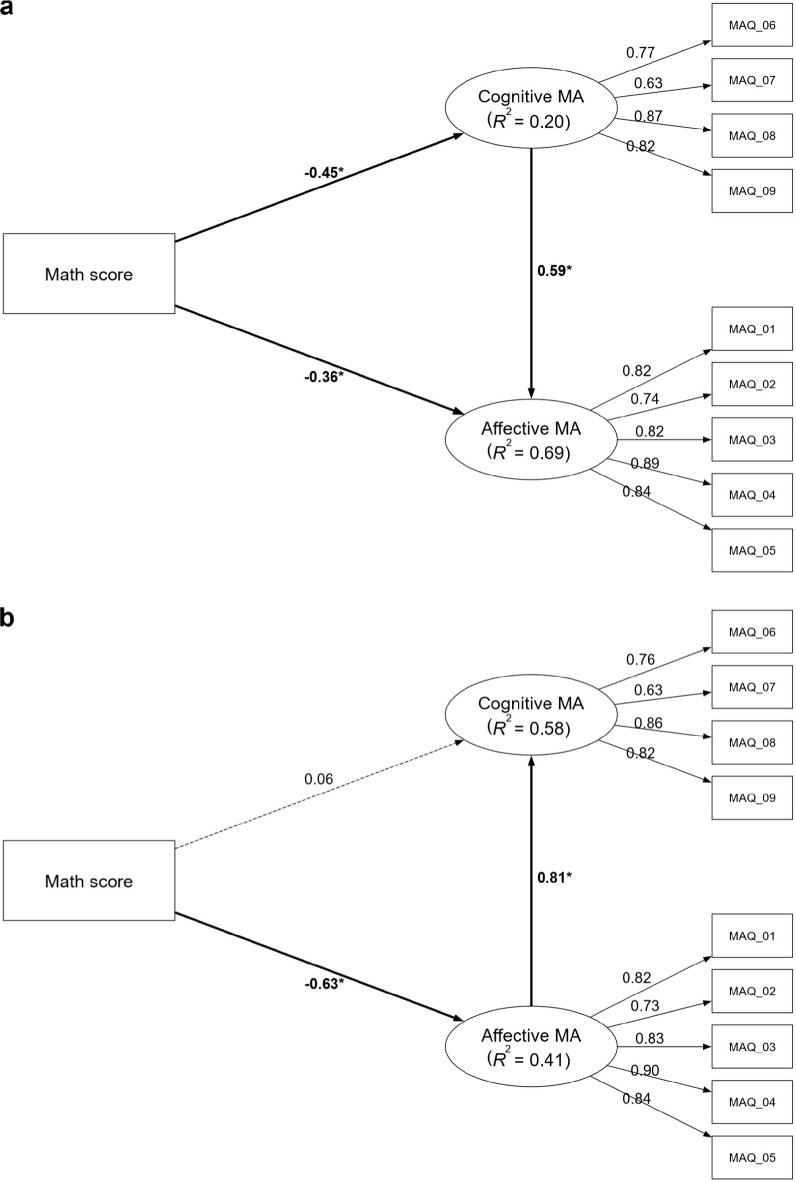
The mediation models of math anxiety in the adult sample. Panel (**a**) displays Model CA (cognitive MA [cMA] → affective MA [aMA]), testing the mediating role of cognitive math anxiety on the relationship between math performance and affective math anxiety. Panel (**b**) displays Model AC (aMA → cMA), testing the mediating role of affective math anxiety. Values on paths represent standardized beta coefficients ($\beta$). The *R*^2^ value under each endogenous construct indicates the proportion of its variance explained by the model. Significant paths are indicated by bold solid lines and marked with an asterisk (*), denoting that the bootstrapped 95% confidence interval (CI) excludes zero. Dashed lines indicate non-significant relationships. Detailed confidence intervals are provided in Supplementary Table S14. All models have controlled for gender, year of exam participation, and language score. MA: math anxiety; MAQ: items of the Math Anxiety Questionnaire.

Psychometric properties in Study 2 mirrored the sound results of Study 1. All loadings, internal consistency, and AVE values were satisfactory (see Fig. 4). Regarding discriminant validity, the HTMT value between the conceptually related affective and cognitive MA constructs was < 0.90, while all other values remained < 0.85. Finally, VIF indices indicated that collinearity was negligible (see Supplemental Results for details).

The mediation analyses replicated the findings of Study 1 with stronger effects. The partial mediation of Model CA (Perf → cMA → aMA) was confirmed (see Fig. 4a), with cognitive MA mediating the performance-to-affective MA link (indirect effect: $\beta$ = -0.27, bootstrap 95% CI [-0.44, -0.14]; total effect: $\beta$ = -0.63, bootstrap 95% CI [-0.78, -0.45]). Notably, the model’s explanatory power for affective MA was substantial (*R*^2^ = 0.69), representing a marked increase from Study 1. The model also explained an acceptable proportion of variance in cognitive MA (*R*^2^ = 0.20). The cognitive-to-affective path remained the dominant mechanism (*f*^2^ = 0.89, large effect), while math performance exerted medium-large direct effects on both cognitive (*f*^2^ = 0.23) and affective (*f*^2^ = 0.30) MA.

Similarly, Model AC’s (Perf → aMA → cMA) full mediation pattern found in Study 1 was consistently replicated (indirect effect: $\beta$ = -0.51, bootstrap 95% CI [-0.72, -0.35]; total effect: $\beta$ = -0.45, bootstrap 95% CI [-0.67, -0.23], see Fig. 4b). The direct path from performance to cognitive MA was again non-significant. This model accounted for moderate variance in both cognitive (*R*^2^ = 0.58) and affective (*R*^2^ = 0.41) MA. The affective-to-cognitive pathway was confirmed as the primary contributor (*f*^2^ = 0.90, large effect), and the direct effect of math performance on affective MA was large (*f*^2^ = 0.60), while its direct link to cognitive MA was negligible (*f*^2^ = 0.004).

**Fig. 5:**
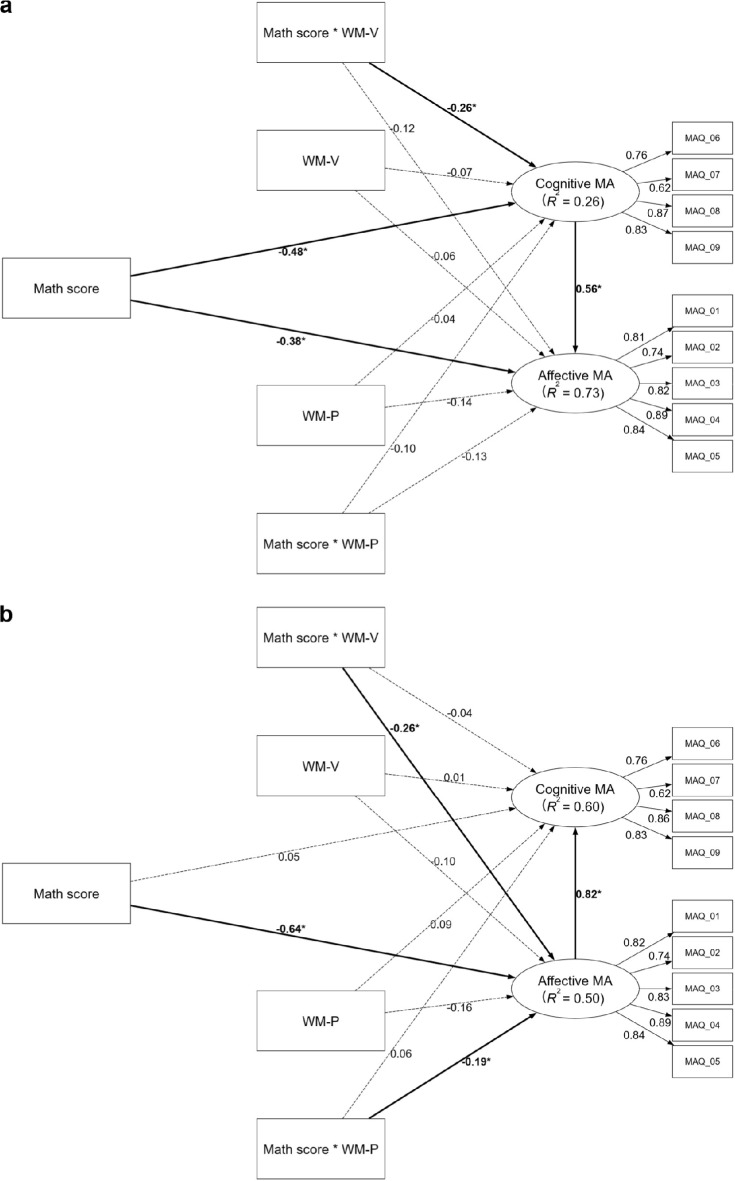
The moderated mediation models of math anxiety in the adult sample. Panel (**a**) displays Model CA (cognitive MA [cMA] → affective MA [aMA]), testing the mediating role of cognitive math anxiety on the relationship between math performance and affective math anxiety. Panel (**b**) displays Model AC (aMA → cMA), testing the mediating role of affective math anxiety. Both models also test the moderating roles of phonological (WM-P) and visuospatial (WM-V) working memory. Paths from interaction terms (e.g., Math score × WM-P) represent moderating effects, while paths from WM constructs represent their direct effects. Values on paths are standardized beta coefficients ($\beta$). The *R*^2^ value indicates the variance explained for each endogenous construct. Significant paths are indicated by bold solid lines and marked with an asterisk (*), denoting that the bootstrapped 95% confidence interval (CI) excludes zero. Dashed lines indicate non-significant relationships. Detailed confidence intervals are provided in Supplementary Table S18. All models have controlled for gender, year of exam participation, and language score. MA: math anxiety; WM-P: working memory (phonological); WM-V: working memory (visuospatial); MAQ: items of the Math Anxiety Questionnaire.

**Fig. 6:**
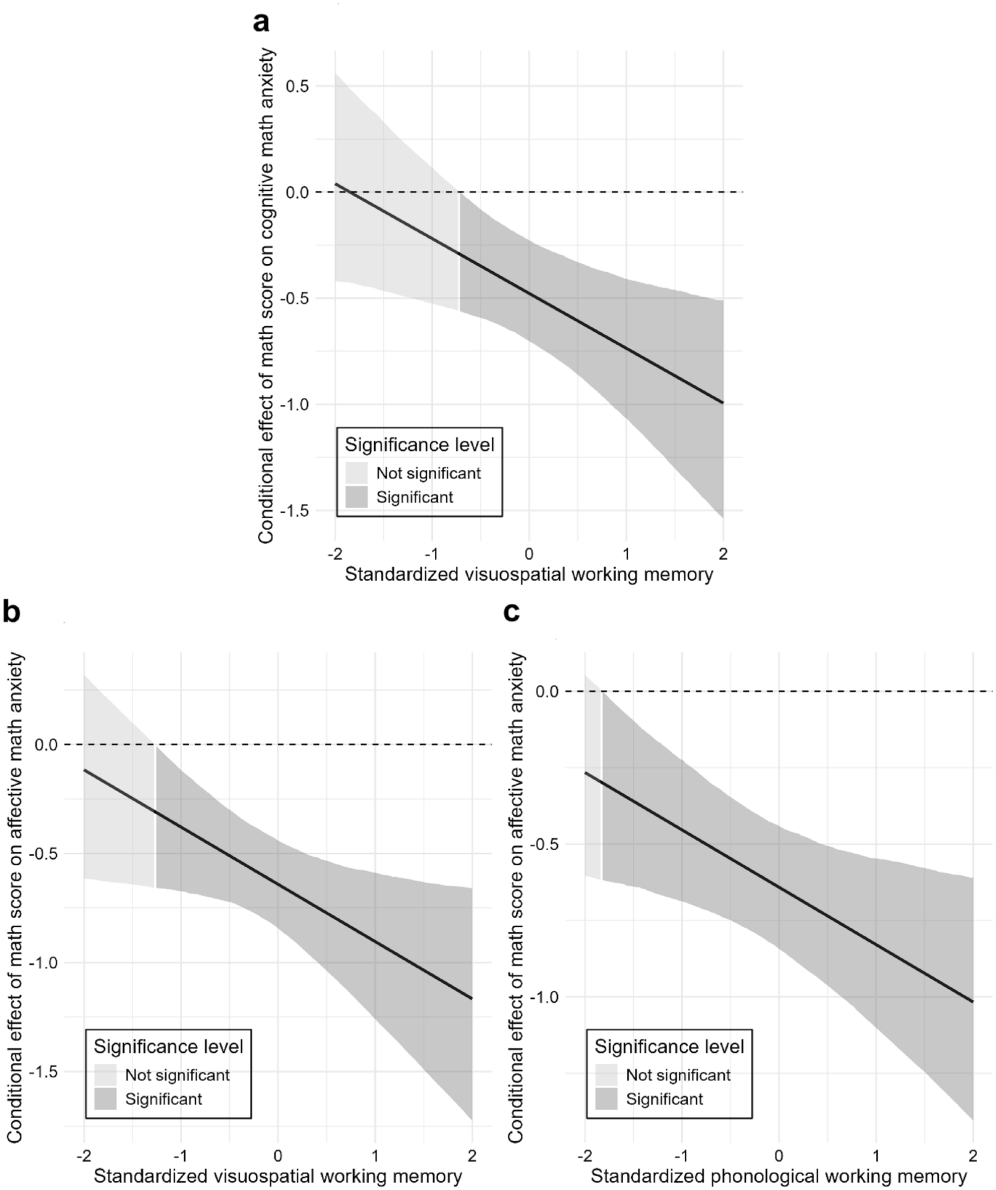
The moderating effects of working memory on the relationship between math performance and math anxiety in the adult sample. The Johnson-Neyman plots illustrate the conditional effect of math performance across standardized levels of working memory. Panel (**a**) shows the moderating effect of visuospatial working memory on cognitive math anxiety, as estimated in Model CA (cognitive math anxiety [cMA] → affective math anxiety [aMA], which tests the mediating role of cognitive anxiety). Panels (b) and (c) show the moderating effects as estimated in Model AC (aMA → cMA, which tests the mediating role of affective anxiety). Specifically, Panel (**b**) displays the effect of visuospatial working memory, whereas Panel (**c**) displays the effect of phonological working memory on affective math anxiety. In all plots, the solid line represents the point estimate of the conditional effect, the shaded area represents the 95% confidence interval, and the dark gray region highlights where the effect is statistically significant.

The moderated mediation analyses provided critical extensions. In a significant departure from Study 1, Moderated Model CA now supported a moderated mediation effect (index of moderated mediation: $\beta$ = -0.14, bootstrap 95% CI [-0.31, -0.03]). While the underlying indirect ($\beta$ = -0.27, bootstrap 95% CI [-0.44, -0.12]) and total ($\beta$ = -0.64, bootstrap 95% CI [-0.84, -0.44]) effects remained significant (see Fig. 5a), this novel moderation effect represents a key finding from the model, which was driven by visuospatial WM moderating the pathway’s first stage (performance-to-cognitive MA; *f*^2^ = 0.07, a very large effect). The negative effect was significant for individuals with medium-to-high visuospatial WM (above -0.71 *SD*, see Fig. 6a). The model’s explanatory power was substantial for affective MA (*R*^2^ = 0.73) and weak for cognitive MA (*R*^2^ = 0.26). Critically, the moderating effect of phonological WM on the performance-to-affective MA path was non-significant, despite the effect size (*f*^2^ = 0.07) being large and identical to that of the significant first-stage visuospatial-WM path. This divergence between magnitude and statistical certainty suggests that estimate of the effect lacked sufficient precision in this sample. Furthermore, no other moderation paths were statistically significant: neither the effect of visuospatial WM on the path to affective MA (*f*^2^ = 0.04, large effect) nor that of the phonological WM on the path to cognitive MA (*f*^2^ = 0.01, medium effect) was statistically significant.

Similarly showing a more complex picture than found in Study 1, Moderated Model AC also supported moderated mediation, with a significant index moderated by both visuospatial ($\beta$ = -0.22, bootstrap 95% CI [-0.48, -0.02]) and phonological WM ($\beta$ = -0.15, bootstrap 95% CI [-0.30, -0.03]). The model’s overall indirect ($\beta$ = -0.53, bootstrap 95% CI [-0.85, -0.33]) and total ($\beta$ = -0.48, bootstrap 95% CI [-0.70, -0.22]) effects also remained significant (see Fig. 5b). This model demonstrated that both WM components significantly moderated the performance-to-affective MA path, and it explained moderate variance in affective (*R*^2^ = 0.50) and cognitive (*R*^2^ = 0.60) MA. The moderation by visuospatial WM was significant (*f*^2^ = 0.10, a very large effect), with the negative link holding for individuals with medium to high visuospatial WM (above -1.26 *SD*, see Fig. 6b). The moderation of phonological WM was also significant (*f*^2^ = 0.07, a very large effect), with the negative link holding for most individuals, becoming non-significant only at very low levels of phonological WM (below -1.82 *SD*, see Fig. 6c). In contrast to findings from Model CA, no statistically significant moderating effects were found for the path to cognitive MA from either the phonological (*f*^2^ = 0.007, small effect) or visuospatial WM (*f*^2^ = 0.005, small effect).

#### Model comparison and predictive power

Consistent with the findings of Study 1, the results of model selection process in Study 2 supported the mutual mediation framework. When affective MA was examined as the outcome variable, the model specifying an indirect pathway via cognitive MA (Model CA: Perf → cMA → aMA, BIC = -51.11) demonstrated a superior fit compared to the alternative model where math performance led directly to affective MA (Model AC: Perf → aMA, BIC = -14.20). Similarly, when cognitive MA served as the outcome variable, the indirect path via affective MA (Model AC: Perf → aMA → cMA, BIC = -31.50) was statistically preferred over the direct path from math performance (Model CA: Perf → cMA, BIC = 5.63). Furthermore, in line with Study 1, these parsimonious mediation models exhibited better fit than their more complex moderated counterparts (Moderated Model CA, BIC = -42.88; Moderated Model AC, BIC = -17.86), reinforcing the consistency of the mediation-only structure across the two age groups sampled.

Following model selection, PLS_predict_ was conducted to assess out-of-sample predictive power, using RMSE as the metric. Model CA showed promising predictive performance for affective MA; its RMSE values (1.06, 1.13, 1.19, 1.29, 1.45) were consistently lower than the LM benchmarks (1.14, 1.18, 1.26, 1.30, 1.50). Model AC also demonstrated high predictive power for cognitive MA, with its RMSE values (1.49, 1.90, 1.39, 1.31) outperforming the LM benchmarks (1.58, 2.12, 1.47, 1.43) across all indicators.

In sum, these findings align with those from Study 1, supporting two distinct mediation models with explanatory value and predictive utility without added complexity.

## Discussion

This research aimed to examine the links between mathematics achievement and MA. Our investigation was guided by two primary objectives: first, to test whether one MA dimension accounts for the link between math performance and the other MA dimension in new developmental stages and a different cultural context, extending the previous work with elementary-school children conducted in a Western culture^23^; and second, to examine how WM resources might change or influence this process. Our findings extend the existing literature by highlighting the consistency of these relationships across different student populations, beyond childhood into adulthood, and offering a more detailed understanding of the potential role of WM.

Extending the observations by Henschel and Roick^23^ with elementary-school students, the current research tested junior and senior high-school students (Study 1) and undergraduate students (Study 2). Evidence from both developmental stages consistently supported two distinct routes linking mathematics achievement to anxiety. Specifically, we found that students’ math performance influences their feelings of tension through their thoughts and worries, and conversely, their thoughts about math are shaped by their emotional reactions to the subject. This pattern suggests that rather than one dimension of MA uniformly preceding the other, the two dimensions—emotional feelings (affective) and worried thoughts (cognitive)—engage in a more dynamic, mutual interplay. This finding was replicated across the adolescent and young adult samples in our study. These findings support predictions derived from Pekrun’s^19,20^ theory, suggesting that lower performance may trigger subsequent changes in either cognitive appraisals or emotional reactions. Our findings thus enrich the Reciprocal Theory of MA by demonstrating that a mutual relationship might exist within the MA construct itself. This underscores that worried thoughts and emotional feelings continuously reinforce each other across the developmental stages we investigated.

We also explored whether working memory (WM) changes how mathematics performance is related to anxiety. Although adding these interaction effects did not substantially improve model fit compared to more parsimonious models (i.e., simpler models without interactions), several patterns emerged. Specifically, in the high-school sample, only phonological, not visuospatial, WM was found to change the strength of the link from math performance to affective MA. This result is consistent with previous findings that negative link from negative math-related affects to math performance is more likely to interfere with the functioning of phonological WM, particularly in earlier developmental stages^53^. We found this moderating effect to be most pronounced among high-school students with low to medium WM capacity, conceptually supporting the account of a “protective buffer” role of WM^28^. Furthermore, while the original theory explains how WM buffers the effects of MA on performance, our findings suggest that a similar mechanism may also operate in the reverse direction: high-WM students might use their cognitive resources to analyze failures and regulate emotions, potentially weakening the link between poor performance and MA; whereas students with lower WM capacity may lack these resources, and when facing situations of underperformance, their excessive worry would strengthen the achievement-to-MA link.

In contrast, among the university students, much more complex patterns emerged. Visuospatial WM influenced the links from performance to both affective and cognitive MA, whereas phonological WM influenced the link to affective MA only, especially in participants with medium and high WM capacity. This pattern suggests that older students are more likely to rely on visuospatial WM strategies—such as mental imagery and multi-step manipulation (e.g., carrying over numbers during mental arithmetic)—when solving math problems^25,54^, consistent with the notion that visuospatial WM is more closely tied to complex mathematical reasoning and problem-solving^55^, but inconsistent with a previous study that suggested the links between MA and WM depended on specific WM task properties, emerging only when manipulation of numbers was involved^56^. Our findings, however, suggest that these influences might be detected even through tasks that do not require students to manipulate or calculate numbers (e.g., remembering patterns or locations). Moreover, these effects were especially evident in participants with medium to high WM capacity, a pattern for which the ‘choking under pressure’ theory has offered a parallel explanation^29^. Although this theory traditionally describes how pressure-induced anxiety impairs the performance of high-WM individuals, its core concept—that greater WM capacity allows for more persistent and distracting negative thoughts (i.e., rumination)—may be applied to the reverse pathway as well: After experiencing situations of underperformance, these high-WM individuals may be more likely to process their worrying thoughts and retain their anxious feelings about previous failures, hence strengthening the achievement-to-MA link.

These findings hold notable theoretical and practical implications. Theoretically, they provide a granular evidence base for a persistent, reciprocal dynamic within MA beyond childhood into adulthood^57^. Practically, this interplay suggests that interventions should be multimodal to be effective^58^. Educators and psychologists might consider combining cognitive-behavioral techniques, such as cognitive restructuring, to modify intrusive thoughts (cognitive MA), with relaxation training or desensitization, to manage physiological tension (affective MA), rather than targeting a single dimension in isolation^59^. Our moderation results also caution that vulnerability to academic setbacks is not limited to one end of the spectrum, potentially affecting students with both relatively low and relatively high cognitive abilities.

However, several limitations of the current research must be considered. First, our research relies on cross-sectional, between-person data (i.e., comparing individuals with different memory capacities rather than tracking changes within the same person over time). While the dual-pathway model was consistently replicated, this reflects structural similarities between groups of adolescents and young adults, and should not be generalized to how these processes unfold within an individual student over time. Also, our data do not permit a direct observation of the real-time process by which anxiety might ‘use up’ mental resources during a task. To confirm these dynamic mental processes, future studies should use within-person designs that track the same individuals over multiple time points or employ experimental tasks, such as dual-task paradigms, to test momentary interactions between MA and WM. Second, although the findings were consistent across our two independent samples (high-school and university students) and align with previous research in a Western context^23^, we cannot claim universal robustness regarding temporal stability or additional cultural contexts without further replication. Third, while participants reported no history of learning disabilities, we did not use objective screening tools to identify students with undiagnosed conditions, such as math learning disability (e.g., dyscalculia) or other neurodevelopmental differences. As these conditions can significantly impact both mathematics performance and anxiety levels, future research should include standardized screening measures to more rigorously control for these potential confounds. Finally, the modest sample sizes limited the statistical power for the moderation analyses, which was likely part of the reason why some statistically significant WM interactions did not translate to improved model fit under the BIC criterion, which favors simpler models unless the added complexity by interactive components provides substantial explanatory power. Future research with a substantially large sample size will be essential in addressing this issue.

In summary, this research advances our understanding of the predictive role of mathematics performance in the development of MA beyond childhood. The results support the relevance of mediating pathways between cognitive and affective MA and, more importantly, reveal that this complex, dual-pathway interplay is a consistent feature across the developmental stages examined. Furthermore, through systematical investigation of WM as a moderator, this research highlights that overall predictive power of models must be considered along with statistical significance of specific predictors. These findings thus provide empirical foundations for designing more effective and multidimensional interventions to mitigate the debilitating effects of MA.

## Supplementary Information

Supplementary Information.

## Data Availability

The datasets for this study are available in the Open Science Framework (OSF) repository, https://osf.io/vwnzj/.
