# Supplementary material for: Mathematics performance predicts cognitive and affective math anxiety through mutual mediation pathways from adolescence onward with potential working memory moderations
Source: Sci Rep. 2026 Mar 29;16:10716. doi: 10.1038/s41598-026-45516-y (PMC13039316; doi:10.1038/s41598-026-45516-y)
Supplement: Supplementary file 1 — Supplementary Information. [file 41598_2026_45516_MOESM1_ESM.pdf]

Mathematics performance predicts cognitive and affective math anxiety through mutual mediation pathways from adolescence onward with potential working memory moderations

Chin-Yuan Chang[1], **Min Hsiao**[1], **and Wen-Chi Chiang**[1,*]

[1]Department of Psychology, National Chung Cheng University, Chiayi 621301, Taiwan (R.O.C.)
[*]psywcc@ccu.edu.tw

Supplemental Results

Study 1

Mediation Models

We used PLS-SEM to test the hypothesized pathways in high-school student sample, controlling for gender, year of exam participation, and language proficiency. The measurement model in Mediation Model CA (math performance [Perf] → cognitive MA [cMA] → affective MA [aMA]) is identical to that in Mediation Model AC (Perf → aMA→ cMA). All indices of internal consistency (i.e., Cronbach's alpha, composite reliability [rho_C], and Dijkstra–Henseler's rho [rho_A]) exceeded the .70 threshold, and all average variance extracted (AVE) values were above .50 (see Table S1) [1].

Discriminant validity was assessed using the heterotrait-monotrait ratio (HTMT) value. For conceptually similar constructs, HTMT values should be below 0.90, and for conceptually distinct constructs, below 0.85. In the present study, all HTMT values were well below the more conservative threshold of 0.85 (see Table S2), indicating strong discriminant validity [2]. Moreover, in both Models CA and AC, all variance inflation factor (VIF) values were below 5, indicating no collinearity issues (see Table S3).

Following the assessment of the measurement model, latent variable correlations were examined, followed by the evaluation of structural model relationships. The latent variable correlation matrix is provided in Table S4. The detailed results of the path analysis, including standardized path coefficients (β) and bootstrapped 95% confidence intervals (CIs) for all structural paths—including direct, indirect, and total effects—are summarized in Table S5.

Construct	Cronbach's alpha	rho_C	AVE	rho_A
Cognitive math anxiety	0.70	0.81	0.52	0.71
Affective math anxiety	0.85	0.89	0.62	0.86

Supplementary Table S1. Internal Consistency Reliability: Mediation Models in the Adolescent Sample

Construct A	Construct B	HTMT Ratio
Math performance	Cognitive math anxiety	0.26
Cognitive math anxiety	Affective math anxiety	0.76
Math performance	Affective math anxiety	0.40

Note. HTMT: Heterotrait-Monotrait Ratio. This ratio assesses discriminant validity. Values below 0.85 (or 0.90 for conceptually similar constructs) indicate that the two constructs are distinct.

Supplementary Table S2. Heterotrait-Monotrait Ratio: Mediation Models in the Adolescent Sample

Predictor Construct	Model CA		Model AC	
	Affective MA	Cognitive MA	Affective MA	Cognitive MA
Year of exam participation	1.52	1.40	1.40	1.51
Language performance	1.34	1.30	1.30	1.33
Gender	1.12	1.07	1.07	1.07
Affective MA				1.28
Cognitive MA	1.22			
Math performance	1.93	1.66	1.66	2.12

Note. MA: math anxiety; Model CA: math performance → cognitive MA → affective MA; Model AC: math performance → affective MA → cognitive MA. Values represent Variance Inflation Factors (VIF). VIF less than 5 indicates no collinearity.

Supplementary Table S3. Variance Inflation Factor: Mediation Models in the Adolescent Sample

Construct	1.	2.	3.	4.	5.	6.
1. Cognitive math anxiety	—	.61***	-.22	.07	.07	-.21
2. Affective math anxiety		—	-.37**	-.01	-.03	-.04
3. Math performance			—	.52***	.41***	-.05
4. Year of exam participation				—	.12	.05
5. Language performance					—	-.24*
6. Gender						—

$N = 65$. * $p < .05$. ** $p < .01$. *** $p < .001$.
Note. Gender was dummy coded (0 = female, 1 = male).

Supplementary Table S4. Latent Variable Correlation Matrix: Mediation Models in the Adolescent Sample

Model	Path	Effect Type	β	95% CI [LL, UL]
Model CA	(Math performance → Cognitive MA → Affective MA)			
	**Cognitive MA → Affective MA**	**Direct**	**0.53**	**[0.30, 0.74]**
	**Math performance → Cognitive MA**	**Direct**	**-0.46**	**[-0.72, -0.21]**
	**Math performance → Affective MA**	**Direct**	**-0.35**	**[-0.66, -0.04]**
	**Math performance → Cognitive MA → Affective MA**	**Indirect**	**-0.25**	**[-0.45, -0.10]**
	**Math performance → Affective MA**	**Total**	**-0.60**	**[-0.86, -0.31]**
	**Exam Year → Cognitive MA**	**Control**	**0.30**	**[0.04, 0.57]**
	Exam Year → Affective MA	Control	0.13	[-0.10, 0.39]
	Language performance → Cognitive MA	Control	0.18	[-0.07, 0.44]
	Language performance → Affective MA	Control	0.07	[-0.17, 0.29]
	Gender → Cognitive MA	Control	-0.20	[-0.42, 0.03]
	Gender → Affective MA	Control	0.07	[-0.13, 0.28]
Model AC	(Math performance → Affective MA → Cognitive MA)			
	**Affective MA → Cognitive MA**	**Direct**	**0.56**	**[0.32, 0.76]**
	Math performance → Cognitive MA	Direct	-0.13	[-0.41, 0.16]
	**Math performance → Affective MA**	**Direct**	**-0.60**	**[-0.86, -0.31]**
	**Math performance → Affective MA → Cognitive MA**	**Indirect**	**-0.33**	**[-0.56, -0.16]**
	**Math performance → Cognitive MA**	**Total**	**-0.46**	**[-0.72, -0.20]**
	Exam Year → Cognitive MA	Control	0.14	[-0.12, 0.38]
	**Exam Year → Affective MA**	**Control**	**0.29**	**[0.01, 0.58]**
	Language performance → Cognitive MA	Control	0.08	[-0.14, 0.32]
	Language performance → Affective MA	Control	0.17	[-0.10, 0.44]
	Gender → Cognitive MA	Control	-0.18	[-0.38, 0.02]
	Gender → Affective MA	Control	-0.04	[-0.28, 0.20]

Note: β = Standardized path coefficient. MA: math anxiety; CI: confidence intervals based on 5,000 bootstrap samples; ; LL = lower limit; UL = upper limit. Bold values indicate that the 95% CI excludes zero.

Supplementary Table S5. Structural Path Estimates: Mediation Models in the Adolescent Sample

Moderated Mediation Models

The quality criteria for the measurement model (i.e., Cronbach's alpha, rho_C, AVE, and rho_A) are identical for both the mediation models and the moderated mediation models, as the introduction of moderator variables did not alter the specification of the original constructs.

All HTMT values were well below the more conservative threshold of 0.85 (see Table S6), indicating strong discriminant validity [2]. In addition, all the VIF values were lower than 5, indicating no collinearity issues (see Table S7).

The latent variable correlation matrix is provided in Table S8. Detailed path analysis results, including β and bootstrapped 95% CIs for all direct, indirect, and total effects, are summarized in Table S9.

Construct A	Construct B	HTMT Ratio
Cognitive math anxiety	Math performance	0.26
Cognitive math anxiety	WM-P	0.13
Cognitive math anxiety	Math performance * WM-P	0.22
Cognitive math anxiety	WM-V	0.11
Cognitive math anxiety	Math performance * WM-V	0.26
Cognitive math anxiety	Affective math anxiety	0.76
Math performance	WM-P	0.41
Math performance	Math performance * WM-P	0.38
Math performance	WM-V	0.21
Math performance	Math performance * WM-V	0.37
Math performance	Affective math anxiety	0.40
WM-P	Math performance * WM-P	0.21
WM-P	WM-V	0.12
WM-P	Math performance * WM-V	0.19
WM-P	Affective math anxiety	0.08
Math performance * WM-P	WM-V	0.14
Math performance * WM-P	Math performance * WM-V	0.32
Math performance * WM-P	Affective math anxiety	0.45
WM-V	Math performance * WM-V	0.59
WM-V	Affective math anxiety	0.25
Math performance * WM-V	Affective math anxiety	0.18

Note. HTMT: Heterotrait-Monotrait Ratio. This ratio assesses discriminant validity. Values below 0.85 (or 0.90 for conceptually similar constructs) indicate that the two constructs are distinct. WM-P: working memory (phonological); WM-V: working memory (visuospatial).

Supplementary Table S6. Heterotrait-Monotrait Ratio: Moderated Mediation Models in the Adolescent Sample

Predictor Construct	Model CA		Model AC	
	Affective MA	Cognitive MA	Affective MA	Cognitive MA
Year of exam participation	1.71	1.60	1.60	1.71
Language performance	1.54	1.47	1.47	1.53
Gender	1.14	1.09	1.09	1.09
Affective math anxiety				1.50
Cognitive math anxiety	1.26			
Math performance	2.60	2.37	2.37	2.67
Math performance * WM-P	1.40	1.39	1.39	1.53
Math performance * WM-V	2.98	2.98	2.98	2.98
WM-P	1.50	1.50	1.50	1.50
WM-V	2.62	2.60	2.60	2.65

Note. MA: math anxiety; Model CA: math performance → cognitive MA → affective MA; Model AC: math performance→ affective MA → cognitive MA; WM-P: working memory (phonological); WM-V: working memory (visuospatial). Values represent Variance Inflation Factors (VIF). VIF less than 5 indicates no collinearity.

Supplementary Table S7. Variance Inflation Factor: Moderated Mediation Models in the Adolescent Sample

Construct	1.	2.	3.	4.	5.	6.	7.	8.
1. Cognitive math anxiety	—	$.61^{***}$	-.22	-.03	.08	.07	.07	-.21
2. Affective math anxiety		—	$-.38^{**}$	-.07	$-.25^{*}$	-.01	-.03	-.04
3. Math performance			—	$.41^{***}$	.21	$.52^{***}$	$.41^{***}$	-.05
4. Phonological working memory				—	.12	$.45^{***}$	$.38^{**}$	.03
5. Visuospatial working memory					—	.17	.02	-.01
6. Year of exam participation						—	.12	.05
7. Language performance							—	$-.24^{*}$
8. Gender								—

$N = 65$. $^{*}p < .05$. $^{**}p < .01$. $^{***}p < .001$.
Note. Gender was dummy coded (0 = female, 1 = male).

Supplementary Table S8. Latent Variable Correlation Matrix: Moderated Mediation Models in the Adolescent Sample

Model	Path	Effect Type	β	95% CI [LL, UL]
Model CA	(Math performance → Cognitive MA → Affective MA)			
	**Cognitive MA → Affective MA**	**Direct**	**0.54**	**[0.32, 0.75]**
	**Math performance → Cognitive MA**	**Direct**	**-0.42**	**[-0.78, -0.06]**
	Math performance → Affective MA	Direct	-0.22	[-0.55, 0.16]
	WM-P → Cognitive MA	Direct	-0.04	[-0.40, 0.24]
	WM-P →Affective MA	Direct	0.03	[-0.17, 0.26]
	Math performance * WM-P → Cognitive MA	Interaction	0.10	[-0.18, 0.32]
	**Math performance * WM-P →Affective MA**	**Interaction**	**0.24**	**[0.02, 0.46]**
	WM-V → Cognitive MA	Direct	0.11	[-0.48, 0.52]
	WM-V →Affective MA	Direct	-0.23	[-0.66, 0.06]
	Math performance * WM-V → Cognitive MA	Interaction	0.06	[-0.56, 0.80]
	Math performance * WM-V →Affective MA	Interaction	-0.02	[-0.46, 0.65]
	**Math performance → Cognitive MA → Affective MA**	**Indirect**	**-0.23**	**[-0.48, -0.03]**
	**Math performance → Affective MA**	**Total**	**-0.45**	**[-0.81, -0.05]**
	Exam Year → Cognitive MA	Control	0.30	[-0.01, 0.63]
	Exam Year → Affective MA	Control	0.11	[-0.11, 0.35]
	Language performance → Cognitive MA	Control	0.22	[-0.06, 0.55]
	Language performance → Affective MA	Control	0.07	[-0.16, 0.32]
	Gender → Cognitive MA	Control	-0.19	[-0.44, 0.06]
	Gender → Affective MA	Control	0.07	[-0.15, 0.27]
Model AC	(Math performance → Affective MA → Cognitive MA)			
	**Affective MA → Cognitive MA**	**Direct**	**0.63**	**[0.41, 0.86]**
	Math performance → Cognitive MA	Direct	-0.14	[-0.48, 0.20]
	**Math performance → Affective MA**	**Direct**	**-0.45**	**[-0.81, -0.05]**
	WM-P → Cognitive MA	Direct	-0.04	[-0.35, 0.18]
	WM-P →Affective MA	Direct	0.01	[-0.25, 0.29]
	Math performance * WM-P → Cognitive MA	Interaction	-0.09	[-0.40, 0.13]
	**Math performance * WM-P →Affective MA**	**Interaction**	**0.29**	**[0.09, 0.51]**
	WM-V → Cognitive MA	Direct	0.21	[-0.22, 0.59]
	WM-V →Affective MA	Direct	-0.17	[-0.75, 0.19]
	Math performance * WM-V → Cognitive MA	Interaction	0.05	[-0.60, 0.56]
	Math performance * WM-V →Affective MA	Interaction	0.02	[-0.52, 0.78]
	**Math performance → Affective MA → Cognitive MA**	**Indirect**	**-0.28**	**[-0.56, -0.03]**
	**Math performance → Cognitive MA**	**Total**	**-0.42**	**[-0.78, -0.05]**
	Exam Year → Cognitive MA	Control	0.12	[-0.17, 0.40]
	**Exam Year → Affective MA**	**Control**	**0.28**	**[0.01, 0.57]**
	Language performance → Cognitive MA	Control	0.09	[-0.17, 0.38]
	Language performance → Affective MA	Control	0.19	[-0.06, 0.48]
	Gender → Cognitive MA	Control	-0.17	[-0.38, 0.05]
	Gender → Affective MA	Control	-0.03	[-0.29, 0.19]

Note: β = Standardized path coefficient. MA: math anxiety; WM-P: working memory (phonological); WM-V: working memory (visuospatial); CI: confidence intervals based on 5,000 bootstrap samples; ; LL = lower limit; UL = upper limit. Bold values indicate that the 95% CI excludes zero.

Supplementary Table S9. Structural Path Estimates: Moderated Mediation Models in the Adolescent Sample

Study2

Mediation Models

Consistent with Study 1, we used PLS-SEM to test the hypothesized pathways in the university student sample, controlling for gender, year of exam participation, and language proficiency. The measurement model in Mediation Model CA (Perf → cMA→ aMA) is identical to that in Mediation Model AC (Perf → aMA→ cMA). All measures demonstrated good internal consistency and convergent validity. All indices of internal consistency (i.e., Cronbach's alpha, rho_C, and rho_A) exceeded the .70 threshold, and all AVE values were above .50 (see Table S10) [1].

Furthermore, the HTMT value between affective and cognitive math anxiety (i.e., conceptually similar constructs) was below 0.90, and all other HTMT values were well below the more conservative threshold of 0.85 (see Table S11), indicating acceptable discriminant validity [2]. In addition, all the VIF values were lower than 5, indicating no collinearity issues (see Table S12).

The structural model relationships were evaluated. The latent variable correlation matrix is provided in Table S13. Detailed path analysis results, including β and bootstrapped 95% CIs for all direct, indirect, and total effects, are summarized in Table S14.

Construct	Cronbach's alpha	rho_C	AVE	rho_A
Cognitive math anxiety	0.78	0.86	0.60	0.82
Affective math anxiety	0.88	0.91	0.68	0.88

Supplementary Table S10. Internal Consistency Reliability: Mediation Models in the Adult Sample

Construct A	Construct B	HTMT Ratio
Math performance	Cognitive math anxiety	0.46
Cognitive math anxiety	Affective math anxiety	0.87
Math performance	Affective math anxiety	0.60

Note. HTMT: Heterotrait-Monotrait Ratio. This ratio assesses discriminant validity. Values below 0.85 (or 0.90 for conceptually similar constructs) indicate that the two constructs are distinct.

Supplementary Table S11. Heterotrait-Monotrait Ratio: Mediation Models in the Adult Sample

Predictor Construct	Model CA		Model AC	
	**Affective MA**	**Cognitive MA**	**Affective MA**	**Cognitive MA**
Year of exam participation	1.07	1.07	1.07	1.14
Language performance	1.09	1.09	1.09	1.09
Gender	1.08	1.06	1.06	1.13
Affective MA				1.70
Cognitive MA	1.25			
Math performance	1.38	1.12	1.12	1.80

Note. MA: math anxiety; Model CA: math performance → cognitive MA → affective MA; Model AC: math performance→ affective MA → cognitive MA. Values represent Variance Inflation Factors (VIF). VIF less than 5 indicates no collinearity.

Supplementary Table S12. Variance Inflation Factor: Mediation Models in the Adult Sample

Construct	1.	2.	3.	4.	5.	6.
1. Cognitive math anxiety	—	$.74^{***}$	$-.42^{***}$	-.08	-.08	-.04
2. Affective math anxiety		—	$-.57^{***}$	-.13	.08	-.17
3. Math performance			—	-.14	.23	.22
4. Year of exam participation				—	-.11	-.18
5. Language performance					—	-.02
6. Gender						—

$N = 64$. $^{*}p < .05$. $^{**}p < .01$. $^{***}p < .001$.
Note. Gender was dummy coded (0 = female, 1 = male).

Supplementary Table S13. Latent Variable Correlation Matrix: Mediation Models in the Adult Sample

Model	Path	Effect Type	β	95% CI [LL, UL]
Model CA	(Math performance → Cognitive MA → Affective MA)			
	**Cognitive MA → Affective MA**	**Direct**	**0.59**	**[0.43, 0.76]**
	**Math performance → Cognitive MA**	**Direct**	**-0.45**	**[-0.67, -0.24]**
	**Math performance → Affective MA**	**Direct**	**-0.36**	**[-0.54, -0.16]**
	**Math performance → Cognitive MA → Affective MA**	**Indirect**	**-0.27**	**[-0.44, -0.14]**
	**Math performance → Affective MA**	**Total**	**-0.63**	**[-0.78, -0.45]**
	Exam Year → Cognitive MA	Control	-0.13	[-0.40, 0.13]
	Exam Year → Affective MA	Control	-0.13	[-0.29, 0.05]
	Language performance → Cognitive MA	Control	0.01	[-0.21, 0.23]
	**Language performance → Affective MA**	**Control**	**0.20**	**[0.03, 0.35]**
	Gender → Cognitive MA	Control	0.04	[-0.21, 0.29]
	Gender → Affective MA	Control	-0.08	[-0.22, 0.06]
Model AC	(Math performance → Affective MA → Cognitive MA)			
	**Affective MA → Cognitive MA**	**Direct**	**0.81**	**[0.63, 1.03]**
	Math performance → Cognitive MA	Direct	0.06	[-0.20, 0.35]
	**Math performance → Affective MA**	**Direct**	**-0.63**	**[-0.78, -0.46]**
	**Math performance → Affective MA → Cognitive MA**	**Indirect**	**-0.51**	**[-0.72, -0.35]**
	**Math performance → Cognitive MA**	**Total**	**-0.45**	**[-0.67, -0.23]**
	Exam Year → Cognitive MA	Control	0.04	[-0.18, 0.25]
	Exam Year → Affective MA	Control	-0.21	[-0.41, 0.01]
	Language performance → Cognitive MA	Control	-0.15	[-0.35, 0.03]
	**Language performance → Affective MA**	**Control**	**0.20**	**[0.01, 0.39]**
	Gender → Cognitive MA	Control	0.09	[-0.08, 0.27]
	Gender → Affective MA	Control	-0.06	[-0.27, 0.16]

Note: β = Standardized path coefficient. MA: math anxiety; CI: confidence intervals based on 5,000 bootstrap samples; ; LL = lower limit; UL = upper limit. Bold values indicate that the 95% CI excludes zero.

Supplementary Table S14. Structural Path Estimates: Mediation Models in the Adult Sample

Moderated Mediation Models

The quality criteria for the measurement model (i.e., Cronbach's alpha, rho_C, AVE, and rho_A) are identical for both the Mediation Model and the Moderated Mediation Model, as the introduction of moderator variables did not alter the specification of the original constructs.

The HTMT value between affective and cognitive math anxiety (i.e., conceptually similar constructs) was below 0.90, and all other HTMT values were well below the more conservative threshold of 0.85 (see Table S15), providing evidence of clear discriminant validity [2]. In addition, all the variance inflation factor (VIF) values were lower than 5, indicating no collinearity issues (see Table S16).

The latent variable correlation matrix is provided in Table S17. Detailed path analysis results, including β and bootstrapped 95% CIs for all direct, indirect, and total effects, are summarized in Table S18.

Construct A	Construct B	HTMT Ratio
Cognitive math anxiety	Math performance	0.46
Cognitive math anxiety	WM-P	0.12
Cognitive math anxiety	Math performance * WM-P	0.07
Cognitive math anxiety	WM-V	0.17
Cognitive math anxiety	Math performance * WM-V	0.18
Cognitive math anxiety	Affective math anxiety	0.87
Math performance	WM-P	0.23
Math performance	Math performance * WM-P	0.19
Math performance	WM-V	0.16
Math performance	Math performance * WM-V	0.12
Math performance	Affective math anxiety	0.60
WM-P	Math performance * WM-P	0.05
WM-P	WM-V	0.02
WM-P	Math performance * WM-V	0.32
WM-P	Affective math anxiety	0.20
Math performance * WM-P	WM-V	0.27
Math performance * WM-P	Math performance * WM-V	0.12
Math performance * WM-P	Affective math anxiety	0.07
WM-V	Math performance * WM-V	0.14
WM-V	Affective math anxiety	0.22
Math performance * WM-V	Affective math anxiety	0.12

Note. WM-P: working memory (phonological); WM-V: working memory (visuospatial). HTMT: Heterotrait-Monotrait Ratio. This ratio assesses discriminant validity. Values below 0.85 (or 0.90 for conceptually similar constructs) indicate that the two constructs are distinct.

Supplementary Table S15. Heterotrait-Monotrait Ratio: Moderated Mediation Models in the Adult Sample

Predictor Construct	Model CA		Model AC	
	**Affective MA**	**Cognitive MA**	**Affective MA**	**Cognitive MA**
Year of exam participation	1.09	1.09	1.08	1.19
Language performance	1.24	1.24	1.09	1.17
Gender	1.11	1.08	1.24	1.26
Affective math anxiety				2.01
Cognitive math anxiety	1.35			
Math performance	1.52	1.21	1.21	2.04
Math performance * WM-P	1.25	1.23	1.23	1.31
Math performance * WM-V	1.25	1.17	1.17	1.29
WM-P	1.18	1.18	1.18	1.23
WM-V	1.19	1.18	1.18	1.20

Note. MA: math anxiety; Model CA: math performance → cognitive MA → affective MA; Model AC: math performance → affective MA → cognitive MA; WM-P: working memory (phonological); WM-V: working memory (visuospatial). Values represent Variance Inflation Factors (VIF). VIF less than 5 indicates no collinearity.

Supplementary Table S16. Variance Inflation Factor: Moderated Mediation Models in the Adult Sample

Construct	1.	2.	3.	4.	5.	6.	7.	8.
1. Cognitive math anxiety	—	.74***	-.42***	-.05	-.12	-.08	-.08	-.04
2. Affective math anxiety		—	-.57***	-.18	-.18	-.13	.08	-.17
3. Math performance			—	.23	.16	-.14	.23	.22
4. Phonological working memory				—	-.02	-.08	.11	.08
5. Visuospatial working memory					—	-.16	-.09	.26
6. Year of exam participation						—	-.11	-.18
7. Language performance							—	-.02
8. Gender								—

$N = 65$. $^{*}p < .05$. $^{**}p < .01$. $^{***}p < .001$.
Note. Gender was dummy coded (0 = female, 1 = male).

Supplementary Table S17. Latent Variable Correlation Matrix: Moderated Mediation Models in the Adult Sample

Model	Path	Effect Type	β	95% CI [LL, UL]
Model CA	(Math performance → Cognitive MA → Affective MA)			
	**Cognitive MA → Affective MA**	**Direct**	**0.56**	**[0.37, 0.75]**
	**Math performance → Cognitive MA**	**Direct**	**-0.48**	**[-0.70, -0.23]**
	**Math performance → Affective MA**	**Direct**	**-0.38**	**[-0.59, -0.16]**
	WM-P → Cognitive MA	Direct	-0.04	[-0.29, 0.18]
	WM-P →Affective MA	Direct	-0.14	[-0.29, 0.07]
	Math performance * WM-P → Cognitive MA	Interaction	-0.10	[-0.34, 0.13]
	Math performance * WM-P →Affective MA	Interaction	-0.13	[-0.29, 0.03]
	WM-V → Cognitive MA	Direct	-0.07	[-0.28, 0.21]
	WM-V →Affective MA	Direct	-0.06	[-0.24, 0.09]
	**Math performance * WM-V → Cognitive MA**	**Interaction**	**-0.26**	**[-0.50, -0.05]**
	Math performance * WM-V →Affective MA	Interaction	-0.12	[-0.32, 0.11]
	**Math performance → Cognitive MA → Affective MA**	**Indirect**	**-0.27**	**[-0.44, -0.12]**
	**Math performance → Affective MA**	**Total**	**-0.64**	**[-0.84, -0.44]**
	Exam Year → Cognitive MA	Control	-0.15	[-0.44, 0.14]
	Exam Year → Affective MA	Control	-0.15	[-0.32, 0.03]
	Language performance → Cognitive MA	Control	0.00	[-0.21, 0.23]
	**Language performance → Affective MA**	**Control**	**0.20**	**[0.03, 0.35]**
	Gender → Cognitive MA	Control	0.02	[-0.27, 0.32]
	Gender → Affective MA	Control	-0.11	[-0.26, 0.03]
Model AC	(Math performance → Affective MA → Cognitive MA)			
	**Affective MA → Cognitive MA**	**Direct**	**0.82**	**[0.62, 1.12]**
	Math performance → Cognitive MA	Direct	0.05	[-0.20, 0.42]
	**Math performance → Affective MA**	**Direct**	**-0.64**	**[-0.84, -0.44]**
	WM-P → Cognitive MA	Direct	0.09	[-0.15, 0.30]
	WM-P →Affective MA	Direct	-0.16	[-0.33, 0.05]
	Math performance * WM-P → Cognitive MA	Interaction	0.06	[-0.17, 0.28]
	**Math performance * WM-P →Affective MA**	**Interaction**	**-0.19**	**[-0.33, -0.03]**
	WM-V → Cognitive MA	Direct	0.01	[-0.16, 0.23]
	WM-V →Affective MA	Direct	-0.10	[-0.30, 0.11]
	Math performance * WM-V → Cognitive MA	Interaction	-0.04	[-0.27, 0.23]
	**Math performance * WM-V →Affective MA**	**Interaction**	**-0.26**	**[-0.49, -0.03]**
	**Math performance → Affective MA → Cognitive MA**	**Indirect**	**-0.53**	**[-0.85, -0.33]**
	**Math performance → Cognitive MA**	**Total**	**-0.48**	**[-0.70, -0.22]**
	Exam Year → Cognitive MA	Control	0.05	[-0.19, 0.28]
	**Exam Year → Affective MA**	**Control**	**-0.24**	**[-0.44, -0.01]**
	Language performance → Cognitive MA	Control	-0.16	[-0.37, 0.03]
	**Language performance → Affective MA**	**Control**	**0.20**	**[0.01, 0.37]**
	Gender → Cognitive MA	Control	0.10	[-0.08, 0.33]
	Gender → Affective MA	Control	-0.10	[-0.31, 0.12]

Note: β = Standardized path coefficient. MA: math anxiety; WM-P: working memory (phonological); WM-V: working memory (visuospatial); CI: confidence intervals based on 5,000 bootstrap samples; ; LL = lower limit; UL = upper limit. Bold values indicate that the 95% CI excludes zero.

Supplementary Table S18. Structural Path Estimates: Moderated Mediation Models in the Adult Sample

References

1. Hair, J. F. Jr., Black, W., Babin, B. & Anderson, R. *Multivariate data analysis.* (Cengage Learning, 2019).

2. Henseler, J., Ringle, C. M. & Sarstedt, M. A new criterion for assessing discriminant validity in variance-based structural equation modeling. *J. Acad. Mark. Sci*. **43**, 115–135 (2015).
